# Retinoic Acid Negatively Impacts Proliferation and MC_TC_ Specific Attributes of Human Skin Derived Mast Cells, but Reinforces Allergic Stimulability

**DOI:** 10.3390/ijms18030525

**Published:** 2017-02-28

**Authors:** Magda Babina, Metin Artuc, Sven Guhl, Torsten Zuberbier

**Affiliations:** Department of Dermatology and Allergy, Charité Universitätsmedizin Berlin, 10117 Berlin, Germany; metin.artuc@charite.de (M.A.); sven.guhl@gmx.de (S.G.); torsten.zuberbier@charite.de (T.Z.)

**Keywords:** mast cell, retinoic acid, skin, proliferation, cell cycle, IgER, tryptase, chymase

## Abstract

The Vitamin-A-metabolite retinoic acid (RA) acts as a master regulator of cellular programs. Mast cells (MCs) are primary effector cells of type-I-allergic reactions. We recently uncovered that human cutaneous MCs are enriched with RA network components over other skin cells. Yet, direct experimental evidence on the significance of the RA-MC axis is limited. Here, skin-derived cultured MCs were exposed to RA for seven days and investigated by flow-cytometry (BrdU incorporation, Annexin/PI, FcεRI), microscopy, RT-qPCR, histamine quantitation, protease activity, and degranulation assays. We found that while MC size and granularity remained unchanged, RA potently interfered with MC proliferation. Conversely, a modest survival-promoting effect from RA was noted. The granule constituents, histamine and tryptase, remained unaffected, while RA had a striking impact on MC chymase, whose expression dropped by gene and by peptidase activity. The newly uncovered *MRGPRX2* performed similarly to chymase. Intriguingly, RA fostered allergic MC degranulation, in a way completely uncoupled from FcεRI expression, but it simultaneously restricted MRGPRX2-triggered histamine release in agreement with the reduced receptor expression. Vitamin-A-derived hormones thus re-shape skin-derived MCs numerically, phenotypically, and functionally. A general theme emerges, implying RA to skew MCs towards processes associated with (allergic) inflammation, while driving them away from the skin-imprinted MC_TC_ (“MCs containing tryptase and chymase”) signature (chymase, MRGPRX2). Collectively, MCs are substantial targets of the skin retinoid network.

## 1. Introduction

Mast cells (MCs), unique elements of the human body [1,2], are of hematopoietic origin, but develop exclusively in target organs, such as the skin, and the intestinal or respiratory tracts, respectively [3,4,5]. In humans, MCs are best understood for their role as effectors of IgE-mediated allergic reactions and in the skin, as intermediaries of pruritus symptoms by interaction of MC derived mediators with sensory nerves [6,7,8]. Accordingly, MCs are numerically increased and/or functionally altered in skin conditions like atopic dermatitis, psoriasis and urticaria [9,10,11].

The skin is not only particularly rich in MCs even in the absence of infection or inflammation [12,13,14], but it is also a firmly established target of Vitamin A derivatives, of which the carboxylic acid form, all-trans retinoic acid (RA), represents the major hormonally active entity.

A tight association between the retinoid system and the skin is underscored by the amounts of RA and its precursors found in this organ and the abundance of proteins involved in RA metabolism and function [15,16], and further substantiated by the broad therapeutic use of retinoids for skin disorders; in fact, retinoid based therapies are inclined towards dermatological conditions [17,18,19,20,21,22,23,24].

So far, skin MCs have not been in the focus of cutaneous RA research. This may reflect the fact that MC purification from skin is costly and laborious and the starting material not widely accessible to researchers. We have overcome this hurdle, and routinely purify MCs from human skin to homogeneity [1,13,25,26].

By interrogating the comprehensive *FANTOM5* (Functional annotation of the mammalian genome 5) expression atlas, which encompasses skin MCs from our laboratory [27], we surprisingly found that it was MCs that expressed the highest levels of the most versatile retinoid receptor subtype (*RARA*, retinoic acid receptor alpha) across the entire atlas [28]. The extensive coverage of the atlas with nearly 1900 libraries from all parts of the body, including ~200 primary cells, as well as time-course series [27,29], implied an important, yet largely overlooked connection between RA and skin MCs. Following up on this theory, we found further evidence through bioinformatics analyses, pinpointing skin MCs as highly enriched with genes pertinent to the retinoid network vis-à-vis other skin cell subsets [28].

These findings spurred further interest in the modulatory effect exerted by RA in MCs from the skin compartment. Focusing on RA’s role in mitogenesis, MC signature characteristics and functional competence, we now report that RA has a profound impact on skin-derived MCs, where it interferes with proliferation and selective attributes of the skin-dwelling MC_TC_ subset, while simultaneously promoting allergic, yet suppressing pseudo-allergic (MRGPRX2-driven) MC degranulation. Collectively, our study substantiates a close connection between skin MCs and the skin RA network and implies MCs as considerable elements in cutaneous retinoid biology under homeostatic and therapeutic conditions.

## 2. Results

### 2.1. RA Counters Proliferation but Has No Negative Impact on the Survival of Human Skin-Derived MCs

Even though classified as terminally differentiated cells, tissue MCs can re-enter the cell cycle if supported by sufficient amounts of stem cell factor (SCF), and this phenomenon also accounts to MCs derived from human skin [30]. Under optimal conditions, the cells require 1–2 weeks to complete one cycle of replication [26,30,31] and express cell cycle regulators like E2F1 and PCNA (proliferating cell nuclear antigen) during this period [1,31].

**Figure 1:**
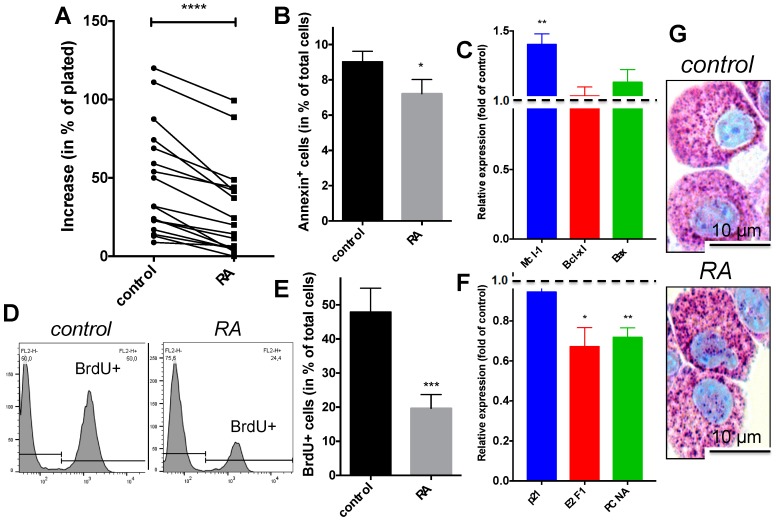
Retinoic acid (RA) inhibits cell cycle progression of skin-derived mast cells (MCs), but also modestly interferes with cell death. Cultured skin-derived MCs (at the proliferative stage) were treated with RA as described in the Experimental section and harvested after 7 days. (**A**) Increase in MC numbers (relative to day 0) in the presence versus absence of RA in a variety of donors, indicated by interconnected dots (*n* = 17); (**B**) Annexin^+^ cells (in % of total cells) after culture with and without RA, mean ± SEM, *n* = 5; (**C**) Expression of apoptosis related genes in RA treated cells normalized to the “no RA” control, mean ± SEM, *n* = 9, dotted line drawn at 1 to highlight control expression (in the absence of RA); (**D**,**E**) BrdU incorporation (added 5 days before harvest), as determined by flow-cytometry, (**D**) representative example, (**E**) mean ± SEM of *n* = 7; (**F**) Expression of cell cycle associated genes in RA treated cells normalized to the “no RA” control, mean ± SEM, *n* = 9; (**G**) Visualization of MCs upon acidic toluidine blue staining reveals no conspicuous changes by RA; note the bright purple granules and the accumulation of cytoplasm, the latter typical of MCs exposed to SCF; (**A**,**B**,**E**) Paired *t*-test, (**C**,**F)** one-sample *t*-test versus control (set as 1). * *p* < 0.05; ** *p* < 0.01, *** *p* < 0.001, **** *p* < 0.0001 versus control.

Here we found that RA counteracted the SCF-driven increment in MC numbers (Figure 1A). Addressing the mechanism, we detected that the proportion of Annexin^+^ (i.e., apoptotic) cells was not increased but even slightly (yet significantly) diminished in the presence of RA (Figure 1B). This was supported by the expression profile of selected genes, where anti-apoptotic Mcl-1 slightly increased, while Bcl-xl as well as pro-apoptotic Bax remained unchanged by RA (Figure 1C).

Therefore, RA did obviously not act by triggering cell death in our system. We next tested whether RA affected cell cycle progression of skin-derived MCs, and found that this was indeed the case. Using BrdU incorporation to track DNA synthesis, an anti-proliferative effect of RA, i.e., interference with SCF-driven cell cycle progression, was easily detected (Figure 1D,E). The anti-proliferative effect was accompanied by changes in selected transcripts associated with the cell cycle. While RA did not influence p21 levels in our system, it diminished PCNA and E2F1 expression (Figure 1F), two genes whose activity correlates with cell cycle progression of skin-derived MCs [1,31].

The microscopic evaluation of toluidine blue stained MCs revealed intact granule architecture in RA treated MCs (Figure 1G), and the cell size likewise remained unaffected in the presence of RA (mean diameter 13.94 ± 0.21 μm for SCF alone, 13.96 ± 0.18 μm for RA), suggesting that SCF-mediated cell growth was normal.

We conclude that the reduced increment in MC numbers in the presence of RA results from interference with SCF-evoked proliferation, but not from reduced viability of non-cycling MCs.

### 2.2. Granule Constituents

MC granules are filled with an array of preformed mediators (especially histamine, heparin, and MC specific proteases) and represent the most salient feature of the lineage (see Figure 1G). Accordingly, transcripts for the granule-associated proteases tryptase and chymase are among the most abundant and specific MC signature genes [1,32,33]. Histamine, on the other hand, mediates many of the clinical symptoms resulting from allergic MC activation and is therefore a major target of anti-allergic medication.

**Figure 2:**
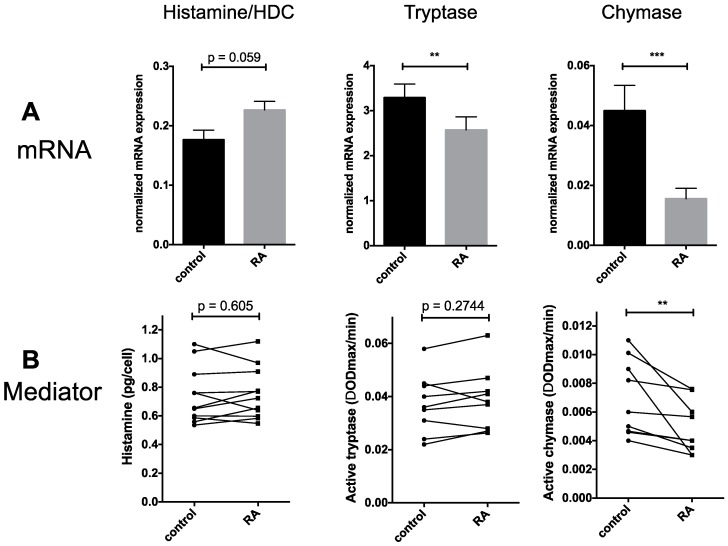
Granule-associated mediators: RA dampens chymase mRNA and chymase activity. (**A**) Relative mRNA expression of histidine decarboxylase (HDC, involved in histamine generation), tryptase and chymase normalized to β actin, respectively, in RA treated and control cells (mean ± SEM, *n* = 9); (**B**) Mediator contents. Histamine was quantified by an auto-analyzer based method, while tryptase and chymase activity were measured by cleavage of specific substrates in RA pretreated and control cells, as described in the Experimental section. Each dot corresponds to one MC preparation and dots representing the same donor are connected. *n* = 11 (histamine) or 9 (tryptase, chymase). ** *p* < 0.01, *** *p* < 0.001 versus control (by paired *t*-test).

We assessed whether RA modulated cellular histamine accumulation and expression of l-histidine decarboxylase (HDC), the enzyme catalyzing the rate-limiting step in histamine formation. Both HDC mRNA and histamine contents remained comparable in cells exposed to RA versus cells kept in SCF alone (Figure 2A,B, left), even though there was a tendency of increased expression in the case of HDC gene expression (Figure 2A, left).

Focusing on MC specific proteases, we found that tryptase mRNA was dampened by RA, whereas tryptase activity remained comparable between the groups (Figure 2A,B, middle). Conversely, chymase mRNA experienced a highly significant drop in the presence of RA down to approximately one third (34.7%) of control expression (Figure 2A, middle). This was accompanied by reduced chymase activity, which showed the same tendency, even though the effect was less pronounced (decrease to 70.8% by mean, and to 66.7% by median of control) (Figure 2B, middle).

Collectively, RA does not impact the preformed mediators histamine and tryptase in our system. Intriguingly, however, there is a particularly clear (negative) effect on MC chymase, a selective marker of the MC_TC_ subcategory, i.e., MCs containing tryptase and chymase (the subset found in the skin over MC_T_ cells, which only contain tryptase and predominate in the lung or gut).

### 2.3. FcεRI Expression and Allergic MC Stimulation

**Figure 3:**
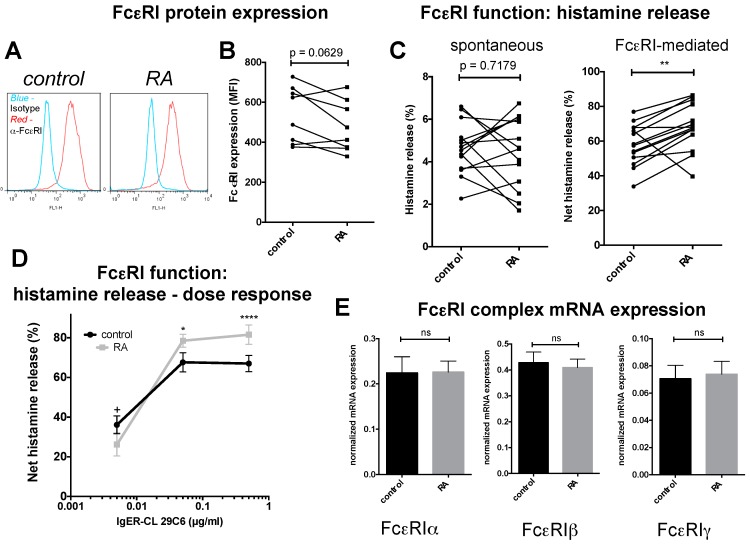
RA reinforces FcεRI-mediated degranulation, but has no effect on the expression of the FcεRI receptor complex. (**A**,**B**) FcεRI cell surface expression by flow-cytometry; (**A**) Representative example, (**B**) cumulative data from *n* = 8 MC preparations; MFI = Mean fluorescence intensity; (**C**) Left, Histamine release in the absence of a specific stimulus (spontaneous) in % of the complete cellular histamine present in the respective MC preparation; right, net histamine release specifically elicited by FcεRI-aggregation (note that spontaneous release, determined for each preparation, was subtracted from the corresponding value after FcεRI-triggering) in RA pretreated and control MCs. *n* = 14; (**B**,**C**) Each dot corresponds to one MC preparation and dots representing the same donor are connected; (**D**) Dose-response of histamine release in RA pretreated and control cells triggered by different concentrations of the IgER-aggregating antibody 29C6, experiment as in (**C**) but using another set of MC preparations and other concentrations, *n* = 7. (**E**) Relative mRNA expression of the three chains forming the FcεRI complex normalized to β actin (mean ± SEM, *n* = 9). * *p* < 0.05, ** *p* < 0.01, **** *p* < 0.0001 significantly higher than control, + *p* < 0.05 significantly lower than control; ns = not significant.

FcεRI, the high affinity receptor for IgE (IgER), represents a classical functional unit of MCs and has been investigated thoroughly for its role in type-I allergic reactions [6,8]. In the present study, cell surface level of FcεRIα remained unchanged in the presence of RA, even though there was a tendency towards downregulated expression (Figure 3A,B). When studying the impact of RA on FcεRI-triggered degranulation responses, we found that histamine release was fostered by prior RA treatment. In fact, a heightened release was detected in 12 out of 14 samples (donors), while one preparation showed the opposite trend and another one no impact from RA. Despite the divergent preparation statistical probability was *p* < 0.01 (Figure 3C, right), which rose to *p* < 0.0001 without the deviating sample (not illustrated). In contrast, blank histamine release (i.e., release in the absence of a specific stimulus) showed no tendency in any direction and both down- and up-regulations were common (Figure 3C, left). Note that the spontaneous release is manifoldly lower than the release elicited by FcεRI aggregation (different scale on the *y*-axis) and also has a greater error rate. In Figure 3C (right), the spontaneous release was already subtracted from the gross histamine release obtained for IgER stimulated cells (i.e., the net values are given in the figure, as detailed in the experimental section). Histamine exteriorization under SCF saturation is very pronounced [26,31], reaching up to 77% of the total cellular histamine content in control cells of the current dataset (Figure 3C). Because of this, there does not seem to be much room for further intensification, and the fact that RA can actually do this and drive degranulation further up seemed quite remarkable. We therefore asked whether RA’s positive impact on IgER-triggered degranulation was dependent on the strength of the signal. Varying the cross-linking agent over a range of 100-fold, we found a clearly supportive effect at the high and intermediate cross-linking strength, confirming the results shown in Figure 3C by an independent set of MC preparations (Figure 3D). At the lowest signal strength, however, RA pre-treatment reduced degranulation, and this resulted in a changed dose-response curve for RA-treated versus control MCs with a steeper increase between 0.005 and 0.05 μg/mL in the former (Figure 3D).

The IgER complex is expressed as a tetramer consisting of three subunits in the composition of αβγ_2_, where the α chain (protruding from the cell surface) is responsible for IgE binding, while the β and γ chains (the former not absolutely required for complex formation) are involved in signal transduction [34]. By quantitative RT-PCR, α, β and γ specific transcripts remained unchanged in the presence of RA (Figure 3E), largely in accordance with the protein expression data depicted in Figure 3A,B. We conclude that RA stimulates allergic MC activation via the FcεRI complex if the cross-linking efficiency is sufficiently pronounced. This occurs without altered expression of the IgER complex, but by reinforced releasability via the IgER complex.

### 2.4. Other MC Selective Traits

**Figure 4:**
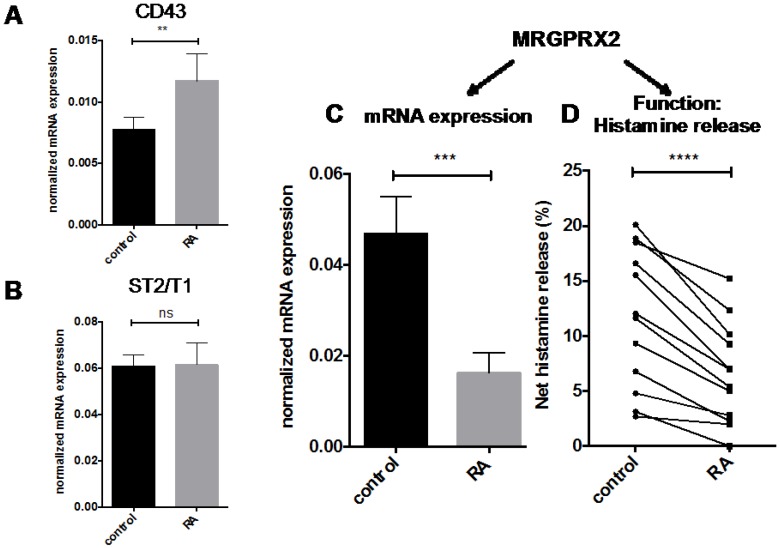
Impact of RA on other genes and MC-restricted processes. Relative mRNA expression, normalized to β actin, for (**A**) the pan-hematopoietic sialoprotein CD43 (the latter used as control of an RA-upregulated gene); (**B**) the IL-33 receptor ST2/T1, and (**C**) the MC-selective receptor MRGPRX2, Mean ± SEM, *n* = 9; (**D**) net histamine release elicited by MRGPRX2 triggering (by use of its agonist compound 48/80 at 10 μg/mL) in RA pretreated and control MCs (analogous to Figure 3C,D). *n* = 12. Each dot corresponds to one MC preparation and dots representing the same donor are connected. ** *p* < 0.01, *** *p* < 0.001, **** *p* < 0.0001 significantly different from control (by paired *t*-test); ns = not significant.

The availability of the comprehensive body-wide expression atlas pinpointed MCs as unique cellular elements of the body and identified additional genes with MC restricted or MC enriched expression. Two examples among the uncovered MC “private genes” are T1/ST2 (*IL1RL1*) and *MRGPRX2* [1], the former encoding the IL-33 receptor [35], the latter a G protein coupled receptor mediating so-called “pseudo-allergic” reactions [36]. Here, we investigated their modulation by RA. CD43 (*SPN*), earlier reported to be an RA-responsive gene in MCs [28,37], served as positive control, and was confirmed to also be promoted by RA in the current study (Figure 4A). ST2/T1 as well as *MRGPRX2* were robustly expressed by MCs (Figure 4B,C showing expression against the highly expressed β actin gene) but not by other cell subsets (like dermal fibroblasts and several cell lines, not depicted), substantiating the results from FANTOM5 [1]. T1/ST2 gene expression remained unaffected in the presence of RA (Figure 4B). In contrast, MRGPRX2 expression substantially diminished after RA treatment down to 34.8% (Figure 4C), thereby duplicating the effect on MC chymase. MRGPRX2 is the long-sought receptor for compound 48/80, a typical MC secretagogue able to degranulate MCs of skin origin [36]. We therefore asked whether its decreased expression in RA-treated cells would be mirrored at the functional level. This was clearly found to be the case, and compound 48/80-triggered degranulation was reduced by RA pretreatment in each MC preparation tested (Figure 4D).

## 3. Discussion

Vitamin A derivatives such as RA have long been known for their wide-range of effects in embryonic development and tissue homeostasis alike, where retinoid receptors impinge on gene expression programs by binding as heterodimers to retinoic acid response elements in target genes to regulate multiple cellular functions [38,39,40].

RA has a particularly profound impact on skin biology. In fact, the skin endogenously produces substantial amounts of RA and its precursors (such as retinol) that contribute to the maintenance of skin homeostasis [16], while therapeutically applied retinoids represent approved (topical or oral) treatments for a wide range of skin disorders including psoriasis, acne, photoaging, non-melanoma skin cancer, cutaneous T-cell lymphomas, ichthyosis, as well as chronic hand eczema, and they even form part of cosmetic products [17,18,19,20,21,22,23,24].

So far, the focus of cutaneous RA research was inclined towards keratinocytes and fibroblasts, while cutaneous MCs have not been considered major participants in the retinoid system of the skin. Moreover, studies that did focus on the retinoid-MC connection rarely employed MCs of skin origin [37,41,42,43,44]. However, MC heterogeneity is a well-known phenomenon, where representatives of the lineage display variability across species, tissues, and microenvironments. It therefore came as a surprise that a cell, which had received fairly little attention with regard to the cutaneous Vitamin A system, turned out a more important partaker than envisaged thus far [28]. This fostered our interest in the impact imposed by RA on MCs in human skin, complementing earlier reports on RA’s modulation of adhesion molecules and selected differentiation markers, performed chiefly on immature MC lines and in vitro generated MC progenitors [37,41,42,43,44].

To gain insight into RA’s modulatory activity in MCs of skin origin, the current study was designed to investigate a reasonable spectrum of processes, encompassing basic cellular functions (like survival and proliferation), as well as MC-specific programs like granule constituent accumulation and allergic as well as pseudo-allergic MC degranulation, with the result that RA indeed impacts functional competence of skin-derived MCs.

We first focused on cell cycle progression and cell viability, two fundamental and tightly regulated processes of every cell subset. There is abundant evidence in the literature demonstrating that RA can either enhance or counter mitogenesis depending on cell type and context. In our system, a considerable anti-proliferative action of RA was detected by BrdU incorporation, and this was reflected by changes in cell cycle regulators like PCNA and E2F1, both of which associated with the proliferative state of human skin-derived MCs [1,31]. In contrast, we did not observe any modulation in p21 expression, a gene frequently up-regulated by RA in a variety of leukemic cells [45,46]. Together, this implies that the mechanisms by which RA inhibits mitogenesis are cell-type specific. The anti-proliferative activity detected here for non-transformed skin-derived MCs is consistent with RA-induced cell cycle arrest in leukemia cells, including cells of the human MC line HMC-1 [47,48], as well as in hematopoietic progenitor-derived MCs [43,44]. The combined evidence now indicates that RA interference with MC proliferation occurs in a manner that is largely independent of the precise nature of the MCs (leukemic versus normal, slowly or rapidly dividing, tissue or precursor-derived). Contrary to the inhibited proliferation, RA had no negative impact on MC survival, where even a modest anti-apoptotic effect was revealed. An uncoupling between proliferation and survival, yet in the opposite direction, was previously reported for myeloid progenitors, where *increased* growth by RA was associated with *decreased* survival [49].

Collectively, in cycling MCs, RA will counter cell increments by decelerating cell cycle progression, while under conditions in which no (major) proliferation occurs, the MC compartment will unlikely experience major numerical changes by RA, or else survival will even be enhanced, as was also evidenced in a previous study [44].

In terms of MC-restricted attributes and processes, one finding was that no conspicuous changes occurred in RA treated MCs on microscopic inspection upon staining with toluidine blue, a MC selective dye (Figure 1G). This applied to cell size and the MC-specific granules alike, suggesting that heparin synthesis and overall granule architecture were preserved in the presence of RA. Assessing granule constituents one by one, the finer level of resolution revealed several additional aspects. Here, histamine and tryptase abundance remained unchanged, while chymase experienced a robust and selective drop.

Considering mRNA and mediator levels simultaneously, we found that while tryptase was resistant to alterations at the activity level, there was significant reduction at the mRNA level. The discordance between tryptase mRNA and the corresponding peptidase activity has been observed previously and applies to MCs across donors [13], MCs skewed by IL-4 [26] as well as MCs exposed to IL-33 [50]. The bottom line from the different studies combined is that the predictive value of tryptase mRNA for the active protein is low. The current study substantiates this notion. In our previous report, histamine levels were positively linked to tryptase activity, suggesting that the amount of histamine in the granule may partially dictate tryptase abundance [13]. This is supported by findings in the mouse, as HDC^−/−^ mice (that do not produce histamine) have profound defects in the accumulation of several MC proteases despite normal mRNA levels [51]. Because histamine levels were unchanged in RA-treated cells, it may be speculated that histamine is the critical component in maintaining the amount of active tryptase.

On the other hand, there is better, but far from absolute concordance between mRNA and protein in the case of chymase, where chymase mRNA typically shows the same tendency as chymase activity [13,26], and this was also detected here. In addition, in our population-based study, chymase activity was also positively associated with histamine abundance (yet the strength of the correlation between histamine and chymase was lower than between histamine and tryptase) [13]. The current data further substantiate that mRNA of these highly lineage-specific traits is a necessary but not sufficient prerequisite for the appearance of the functional peptidase products, and that other variables are important, one of which may be the abundance of histamine. Notwithstanding, the chymase gene may serve as a prototypical gene to establish the molecular background by which MCs in the skin acquire their selective MC_TC_ signature and how RA interferes with this process.

Our data may also provide some interpretation on a previous study, in which MCs were enumerated in human skin exposed to topical RA treatment for up to six weeks [44]. In that work, Hjertson and colleagues found an increase in MC_T_ (“MCs containing only tryptase”) MCs in the skin treated with RA cream (which are virtually absent from the skin under normal conditions), while no increase was found for the chymase containing MC_TC_ subset. Our current findings offer an explanation, namely that the de novo appearance of MC_T_ MCs in RA treated skin may result from the down-regulated chymase expression, becoming undetectable to immunohistochemistry. The increase in MCs (of the MC_T_ category) after RA treatment implies that RA has a survival-promoting role in MCs embedded in their natural habitat, which would also be compatible with the present study.

Addressing RA’s impact on the most thoroughly investigated and clinically relevant MC process, i.e., allergic stimulation, the current study revealed that RA unexpectedly, but discernibly promoted degranulation responses triggered by FcεRI cross-linking. Of note, there was a simultaneous tendency towards *reduced* FcεRI expression together with the *increased* FcεRI functionality. This finding adds to the growing appreciation that IgER expression and IgER-triggered degranulation represent uncoupled qualities of MCs ([13] and references therein). Taken together, RA fosters allergic degranulation by a route independent of enhanced FcεRI expression. The process of FcεRI-elicited exocytosis is highly complex and involves not only numerous signaling intermediates, but also a diverse set of accessory proteins which organize traffic, priming, tethering and docking of secretory vesicles, such as members of the Rab, Munc13, and Munc18 families, and especially Ca^2+^ sensors like synaptotagmins and other Ca^2+^ acceptors [52]. RA may influence several of these molecules in different directions, which would be compatible with the finding that at low cross-linking efficiency, inhibition of FcεRI-elicited exocytosis by RA prevails (Figure 3D). From an allergy-centered view the globally rather increased propensity of RA-exposed MCs to exocytosis implies a pro-allergic role of RA at the level of the skin MC, at least in situations in which specific IgE and allergen are intermediate to abundant.

A rather pro-allergic or generally pro-inflammatory function of RA in skin MCs is further supported by our previous observation of strengthened cytokine responses in response to RA [28], and earlier studies showing increased expression of adhesion molecules in (mainly leukemic) MCs [37,41,42]. This view also fits the finding of RA-mediated attraction of T cells not only to mucosal tissues but also to inflamed sites, supportive of a rather inflammation-promoting or -maintaining function of RA [53]. Collectively, the combined data imply that the overall effect of RA on skin MCs is highly gene and process-specific. While MCs seem to adopt a more inflammatory role in the presence of RA, RA apparently drives MCs away from their cutaneous signature. This is based on the finding that RA dampens the “classical” marker of the MC_TC_ subset, i.e., MC chymase. In fact, chymase is a strong criterion of skin MC maturity, because other lineage attributes (including FcεRI and HDC) are also expressed in more immature MCs [54]. This may signify a partially dedifferentiating effect of RA on MCs away from the phenotype imprinted by the surrounding dermal compartment. In further agreement with this notion, RA has a negative impact on MRGPRX2 expression, the receptor for several neuropeptides as well as pseudo-allergic reactions (the latter triggered by a variety of drugs) [36,55]. In fact, using the MRGPRX2 agonist compound 48/80, we directly demonstrate that RA blunts responses to this MC_TC_-related G protein coupled receptor, matching RA’s impact on MRGPRX2 expression (Figure 4D). Like MC chymase, MRGPRX2 seems to be confined to MCs in skin tissue, as MCs of mucosal tissues, including lung (a prototypical member of the MC_T_ type) lack discernible MRGPRX2 expression [56,57].

From a degranulation standpoint, our study revealed an opposite effect of RA on the two major routes of MC degranulation, namely the allergic and the pseudo-allergic pathway. Through a series of elegant experiments, it was recently reported that the two pathways differ quite profoundly regarding signaling prerequisites, dynamics, and granule characteristics [58]. While IgER-triggered degranulation is delayed and shows a progressive pattern, MRGPRX2-mediated degranulation is rapid and associated with a quick and transient peak of intracellular calcium that is rapidly followed by secretion of individual secretory granules. The IgER-elicited pattern depends on IKK-β activation and SNAP23/STX4 complex formation, processes which are implicated in organizing the so-called compound exocytosis, in which several granules fuse together [58]. We show that RA differentially affects the pathways, favoring allergic degranulation via IgER-triggering, while simultaneously inhibiting the pseudo-allergic route.

Together, RA has pleiotropic effects on MCs and seems to operate by different routes in the lineage, on one hand by increasing several immune functions, in which MCs participate, but which are not restricted to the lineage, such as generation of pro-inflammatory cytokines [28], but on the other by reducing the most specific genes and processes of the skin-associated MC_TC_ type.

It may seem counterintuitive that a molecule like RA with its deep connection to skin physiology would counter the expression of MC lineage markers that are skin-specific. It is likely, though, that the expression of each biomolecule is a finely tuned process consisting of positive and negative regulatory circuits. So, even though the skin habitat does provide a MC_TC_-friendly environment, RA may constitute a counter-balancing regulator in this setting. Of note, both chymase and MRGPRX2 are still expressed in skin-derived MCs even after exposure to an RA-rich microenvironment, while they are entirely absent from the mucosa of the intestinal and respiratory tract, respectively. It therefore seems unlikely that the lack of MC_TC_ marker expression in mucosal tissues primarily stems from the presence of RA (even though RA plays a critical role in mucosal immunity and can redirect immune cells to the gut [53,59]), but rather that the constellation of transcription factors and epigenetic attributes may be favorable in the skin over mucosal tissues to permit chymase and MRGPRX2 transcription in the first place. Revealing the molecular prerequisites behind MC_TC_ specific features is actually an important future challenge in the MC field, and the observations made in this study may actually help in identifying the mechanistic details.

Other important future directions will include the dissection of RA-mediated responses in skin MCs in different microenvironments, which by themselves shift MC phenotypes (e.g., IL-4 and IL-33 [26,50]), because it is clear from studies on hematopoietic stem cells that diverse and even opposing effects can occur in dependence of the growth factors present [38,49]. Of note, the adaptive immune system is under substantial influence from RA. For example, RA can skew T helper cell subsets, in particular by supporting Foxp3+ regulatory T cell populations, while simultaneously suppressing Th17 cells [53,59], and RA imprints gut homing in these subsets [53,59]. RA can also modify Th1/Th2 subsets [53], and impact on B cell mediated immunity by fostering antibody-secreting cells producing the IgA isotype [60]. Considering that MCs interact with T cells and are influenced by their cytokines, MCs in the skin may not only be influenced directly (as is the focus of the current report), but also indirectly via RA’s effects on the adaptive immune system. It will therefore be of quite some interest to study the effect of RA on MCs embedded in skin equivalents like the organotypic co-culture skin model developed and refined by our group over many years [61,62] to comprehend RA’s role in MC communication with other resident skin cells (fibroblasts, keratinocytes), as well as other immune constituents like T cells.

While the complexity of RA activity in the stem cell compartment has been recognized and cataloged [38], we are at the beginning of dissecting RA’s impact on the MC lineage, a cell enriched with retinoid network components [28] that shares features with hematopoietic stem cells [1]. The current study provides further evidence that MCs are indeed relevant targets of the retinoid network and should be considered in research of cutaneous retinoid biology.

## 4. Experimental Section

### 4.1. Isolation and Culture of Human Skin MCs

MCs were isolated from human breast skin as described [25] with slight modifications given in recent publications [1,31,63]. The skin was obtained from cosmetic breast reduction surgeries, with the informed consent of the patients and approval by the university ethics committee. The experiments were conducted according to the Declaration of Helsinki Principles. Skin-derived MCs were expanded in basal Iscove’s Medium (with 10% FCS, 100 U/mL Penicillin, 100 μg/mL Streptomycin, 2 mM l-Glutamine, 1% nonessential amino acid solution, all from Biochrom, Berlin, Germany), and stem cell factor (SCF) at 100 ng/mL (Peprotech, Hamburg, Germany), as described [26], as a modification of a previously published procedure [30]. The cells were used for the experiments described herein after 3–5 weeks, i.e., at a stage when proliferation is detectable in MCs from all donors [31].

### 4.2. MC Treatment

MCs were kept at 5 × 10^5^/mL in the presence or absence of RA (Sigma-Aldrich, Taufkirchen, Germany) at 100 nM for a total of 7 days. RA was provided on days 0, 2, and 4, while SCF (100 ng/mL) was provided on days 0 and 4 to maintain optimal growth, proliferation, and survival. MCs were automatically counted by the CASY-TTC and their mean diameter was recorded (Innovatis/Casy Technology, Reutlingen, Germany) [26,31,63].

### 4.3. Toluidine-Blue Staining and Microscopy

MC granules were specifically stained with acidic toluidine-blue (0.1% in 0.5 N HCl), and stained MCs were photographed using a Zeiss Axiovert 10 microscope and a Canon power shot-A620 digital camera, as described [31,63]. The original magnification was 400×.

### 4.4. Annexin-FITC/PI Staining

MCs were stained by the Annexin V-FITC Apoptosis detection kit (eBioscience, San Diego, CA, USA) according to the manufacturer’s instructions and then analyzed by flow-cytometry on a Facscalibur flow-cytometer device (BD Biosciences, Heidelberg, Germany). Annexin-V/PI-double-negative cells were considered alive, while Annexin-V-positive cells were considered apoptotic (Annexin-V-single positive—early apoptotic; Annexin-V-/PI-double positive—late apoptotic), as per instructions.

### 4.5. BrdU Incorporation

DNA duplication was assessed with the BrdU flow kit (BD Pharmingen, Heidelberg, Germany) according to the vendor’s instructions and as described previously for skin-derived MCs [30,31]. In brief, MCs were kept in the presence (or absence for control) of 10 μM BrdU for 5 days prior to harvest and assayed for incorporation of BrdU using anti-BrdU-FITC and flow-cytometry.

### 4.6. Reverse Transcription-Quantitative PCR (RT-qPCR)

RT-qPCR was performed using optimized conditions and primer pairs described elsewhere [13,31,64]. Values were normalized to the housekeeping gene β-actin.

### 4.7. FcεRI Surface Expression

Flow-cytometry was performed as described [13,31]. The antibody used was anti-human FcεRIα-PE at 2.5 μg/mL (clone AER-37, eBioscience, San Diego, CA, USA); mouse IgG2b-PE (clone eBWG2b, eBioscience) served as isotype control.

### 4.8. Histamine Quantitation

Histamine was quantitated by an automated fluorescence method using an autoanalyzer (Borgwald Technik, Hamburg, Germany), exactly as described [13,31]. All determinations were performed in triplicate and histamine concentrations were calculated with reference to a standard curve.

### 4.9. Histamine Release Assay

MCs were tested for degranulation responses (histamine release) following FcεRI-aggregation (using the anti-FcεRIα-Ab 29C6 at 0.25 μg/mL for single-dose or 0.005, 0.05 and 0.5 μg/mL for the dose-response experiments), compound 48/80 at 10 μg/mL (Sigma-Aldrich) or no stimulus for spontaneous release. The assays were performed exactly as described and the net histamine release (%) was calculated as [(stimulated release − spontaneous release)/complete histamine present in the MC preparation] × 100 [13,26,31].

### 4.10. MC Protease Activity

Tryptase and chymase activities were quantified according to our routine protocols [13,31,54] established upon previous publications [65].

### 4.11. Statistics

Differences between groups were assessed by paired or ratio paired *t*-test, or (when not normally distributed) by Wilcoxon matched-pairs signed rank test with the program GraphPad-Prism, San Diego, CA, USA. *p* < 0.05 was considered statistically significant. The number of independent experiments performed for each assay is given in the respective figure legends as *n*.

## 5. Conclusions

The study supports the theory that MCs in human skin are highly relevant targets of Vitamin A metabolites and that RA executes a program in these cells that is oriented towards more general pan-hematopoietic and inflammatory programs while minimizing processes selective for the MC_TC_ subset that prevails in human skin. We expect that transcriptional and functional programs of MCs may experience other profound modulations by RA, calling for a need of further research in the future. Given that retinoids act as important developmental switches, and that retinoid receptors regulate a plethora of genes, the accumulation of retinoid network components in MCs, as recently revealed [28], is of relevance to fields as diverse as vitamin A research, MC biology, and skin (patho-)physiology alike.

## References

[1] Motakis E., Guhl S., Ishizu Y., Itoh M., Kawaji H., de Hoon M., Lassmann T., Carninci P., Hayashizaki Y., Zuberbier T. (2014). Redefinition of the human mast cell transcriptome by deep-CAGE sequencing. Blood.

[2] Babina M., Motakis E., Zuberbier T. (2014). Mast cell transcriptome elucidation: What are the implications for allergic disease in the clinic and where do we go next?. Expert Rev. Clin. Immunol..

[3] Kitamura Y., Oboki K., Ito A. (2007). Development of mast cells. Proc. Jpn. Acad. Ser. B Phys. Biol. Sci..

[4] Metcalfe D.D. (2008). Mast cells and mastocytosis. Blood.

[5] Gilfillan A.M., Beaven M.A. (2011). Regulation of mast cell responses in health and disease. Crit. Rev. Immunol..

[6] Brown J.M., Wilson T.M., Metcalfe D.D. (2008). The mast cell and allergic diseases: Role in pathogenesis and implications for therapy. Clin. Exp. Allergy.

[7] Yosipovitch G., Papoiu A.D. (2008). What causes itch in atopic dermatitis?. Curr. Allergy Asthma Rep..

[8] Galli S.J., Tsai M. (2012). IgE and mast cells in allergic disease. Nat. Med..

[9] Kawakami T., Ando T., Kimura M., Wilson B.S., Kawakami Y. (2009). Mast cells in atopic dermatitis. Curr. Opin. Immunol..

[10] Harvima I.T., Nilsson G., Suttle M.M., Naukkarinen A. (2008). Is there a role for mast cells in psoriasis?. Arch. Dermatol. Res..

[11] Ferrer M., Kaplan A.P. (2007). Progress and challenges in the understanding of chronic urticaria. Allergy Asthma Clin. Immunol..

[12] Bretterklieber A., Beham-Schmid C., Sturm G.J., Berghold A., Brezinschek R., Aberer W., Aberer E. (2015). Anaphylaxis with clonal mast cells in normal looking skin—A new entity?. Allergy.

[13] Babina M., Guhl S., Artuc M., Trivedi N.N., Zuberbier T. (2016). Phenotypic variability in human skin mast cells. Exp. Dermatol..

[14] Dwyer D.F., Barrett N.A., Austen K.F. (2016). Immunological Genome Project Consortium. Expression profiling of constitutive mast cells reveals a unique identity within the immune system. Nat. Immunol..

[15] Fisher G.J., Voorhees J.J. (1996). Molecular mechanisms of retinoid actions in skin. FASEB J..

[16] Mihaly J., Gamlieli A., Worm M., Ruhl R. (2011). Decreased retinoid concentration and retinoid signalling pathways in human atopic dermatitis. Exp. Dermatol..

[17] Varani J., Fisher G.J., Kang S., Voorhees J.J. (1998). Molecular mechanisms of intrinsic skin aging and retinoid-induced repair and reversal. J. Investig. Dermatol. Symp. Proc..

[18] Appa Y. (1999). Retinoid therapy: Compatible skin care. Skin Pharmacol. Appl. Skin Physiol..

[19] De Graaf Y.G., Euvrard S., Bouwes Bavinck J.N. (2004). Systemic and topical retinoids in the management of skin cancer in organ transplant recipients. Dermatol. Surg..

[20] Zhang C., Duvic M. (2006). Treatment of cutaneous T-cell lymphoma with retinoids. Dermatol. Ther..

[21] Lee C.S., Li K. (2009). A review of acitretin for the treatment of psoriasis. Expert Opin. Drug Saf..

[22] Digiovanna J.J., Mauro T., Milstone L.M., Schmuth M., Toro J.R. (2013). Systemic retinoids in the management of ichthyoses and related skin types. Dermatol. Ther..

[23] Mukherjee S., Date A., Patravale V., Korting H.C., Roeder A., Weindl G. (2006). Retinoids in the treatment of skin aging: An overview of clinical efficacy and safety. Clin. Interv. Aging.

[24] Kumari V., Timm K., Kuhl A.A., Heine G., Worm M. (2016). Impact of systemic alitretinoin treatment on skin barrier gene and protein expression in patients with chronic hand eczema. Br. J. Dermatol..

[25] Babina M., Guhl S., Starke A., Kirchhof L., Zuberbier T., Henz B.M. (2004). Comparative cytokine profile of human skin mast cells from two compartments—Strong resemblance with monocytes at baseline but induction of IL-5 by IL-4 priming. J. Leukoc. Biol..

[26] Babina M., Guhl S., Artuc M., Zuberbier T. (2016). IL-4 and human skin mast cells revisited: Reinforcement of a pro-allergic phenotype upon prolonged exposure. Arch. Dermatol. Res..

[27] Forrest A.R., Kawaji H., Rehli M., Baillie J.K., de Hoon M.J.L., Haberle V., Lassmann T., Kulakovskiy I.V., Lizio M., Itoh M. (2014). A promoter-level mammalian expression atlas. Nature.

[28] Babina M., Guhl S., Motakis E., Artuc M., Hazzan T., Worm M., Forrest A.R., Zuberbier T. (2015). Retinoic acid potentiates inflammatory cytokines in human mast cells: Identification of mast cells as prominent constituents of the skin retinoid network. Mol. Cell. Endocrinol..

[29] Arner E., Daub C.O., Vitting-Seerup K., Andersson R., Lilje B., Drablos F., Lennartsson A., Ronnerblad M., Hrydziuszko O., Vitezic M. (2015). Transcribed enhancers lead waves of coordinated transcription in transitioning mammalian cells. Science.

[30] Guhl S., Artuc M., Neou A., Babina M., Zuberbier T. (2011). Long-term cultured human skin mast cells are suitable for pharmacological studies of anti-allergic drugs due to high responsiveness to fcepsilonri cross-linking. Biosci. Biotechnol. Biochem..

[31] Guhl S., Neou A., Artuc M., Zuberbier T., Babina M. (2014). Skin mast cells develop non-synchronized changes in typical lineage characteristics upon culture. Exp. Dermatol..

[32] Caughey G.H. (2007). Mast cell tryptases and chymases in inflammation and host defense. Immunol. Rev..

[33] Pejler G., Abrink M., Ringvall M., Wernersson S. (2007). Mast cell proteases. Adv. Immunol..

[34] Potaczek D.P., Kabesch M. (2012). Current concepts of IgE regulation and impact of genetic determinants. Clin. Exp. Allergy.

[35] Saluja R., Zoltowska A., Ketelaar M.E., Nilsson G. (2016). IL-33 and thymic stromal lymphopoietin in mast cell functions. Eur. J. Pharmacol..

[36] McNeil B.D., Pundir P., Meeker S., Han L., Undem B.J., Kulka M., Dong X. (2015). Identification of a mast-cell-specific receptor crucial for pseudo-allergic drug reactions. Nature.

[37] Babina M., Weber S., Henz B.M. (1997). Cd43 (leukosialin, sialophorin) expression is differentially regulated by retinoic acids. Eur. J. Immunol..

[38] Purton L.E. (2007). Roles of retinoids and retinoic acid receptors in the regulation of hematopoietic stem cell self-renewal and differentiation. PPAR Res..

[39] Rochette-Egly C., Germain P. (2009). Dynamic and combinatorial control of gene expression by nuclear retinoic acid receptors (RARs). Nucl. Recept. Signal..

[40] Gudas L.J., Wagner J.A. (2011). Retinoids regulate stem cell differentiation. J. Cell. Physiol..

[41] Babina M., Weber S., Henz B.M. (1997). Retinoic acids and dexamethasone alter cell-surface density of β2-integrins and ICAM-1 on human leukemic (HMC-1) mast cells. Arch. Dermatol. Res..

[42] Babina M., Mammeri K., Henz B.M. (2001). Retinoic acid up-regulates myeloid ICAM-3 expression and function in a cell-specific fashion—Evidence for retinoid signaling pathways in the mast cell lineage. J. Leukoc. Biol..

[43] Kinoshita T., Koike K., Mwamtemi H.H., Ito S., Ishida S., Nakazawa Y., Kurokawa Y., Sakashita K., Higuchi T., Takeuchi K. (2000). Retinoic acid is a negative regulator for the differentiation of cord blood-derived human mast cell progenitors. Blood.

[44] Hjertson M., Kivinen P.K., Dimberg L., Nilsson K., Harvima I.T., Nilsson G. (2003). Retinoic acid inhibits in vitro development of mast cells but has no marked effect on mature human skin tryptase- and chymase-positive mast cells. J. Investig. Dermatol..

[45] Liu M., Iavarone A., Freedman L.P. (1996). Transcriptional activation of the human *p21(WAF1/CIP1)* gene by retinoic acid receptor. Correlation with retinoid induction of U937 cell differentiation. J. Biol. Chem..

[46] Lavelle D., Chen Y.H., Hankewych M., Desimone J. (1999). Inhibition of myeloma cell growth by all-trans retinoic acid is associated with upregulation of p21WAF1 and dephosphorylation of the retinoblastoma protein. Leuk. Lymphoma.

[47] Alexandrakis M.G., Kyriakou D.S., Seretakis D., Boucher W., Letourneau R., Kempuraj D., Theoharides T.C. (2003). Inhibitory effect of retinoic acid on proliferation, maturation and tryptase level in human leukemic mast cells (HMC-1). Int. J. Immunopathol. Pharmacol..

[48] Nilsson G., Blom T., Kusche-Gullberg M., Kjellen L., Butterfield J.H., Sundstrom C., Nilsson K., Hellman L. (1994). Phenotypic characterization of the human mast-cell line HMC-1. Scand. J. Immunol..

[49] Douer D., Ramezani L., Parker J., Levine A.M. (2000). All-trans-retinoic acid effects the growth, differentiation and apoptosis of normal human myeloid progenitors derived from purified CD34+ bone marrow cells. Leukemia.

[50] Babina M., Guhl S., Artuc M., Zuberbier T. (2017). The yin and yang of IL-33 in human skin mast cell biology—Reinforcement of proliferation and histamine synthesis combined with profound decline in immunological and non-immunological histamine releasability. Nutrients.

[51] Ohtsu H., Tanaka S., Terui T., Hori Y., Makabe-Kobayashi Y., Pejler G., Tchougounova E., Hellman L., Gertsenstein M., Hirasawa N. (2001). Mice lacking histidine decarboxylase exhibit abnormal mast cells. FEBS Lett..

[52] Blank U., Madera-Salcedo I.K., Danelli L., Claver J., Tiwari N., Sánchez-Miranda E., Vázquez-Victorio G., Ramírez-Valadez K.A., Macias-Silva M., González-Espinosa C. (2014). Vesicular trafficking and signaling for cytokine and chemokine secretion in mast cells. Front. Immunol..

[53] Bono M.R., Tejon G., Flores-Santibañez F., Fernandez D., Rosemblatt M., Sauma D. (2016). Retinoic acid as a modulator of T cell immunity. Nutrients.

[54] Guhl S., Babina M., Neou A., Zuberbier T., Artuc M. (2010). Mast cell lines HMC-1 and LAD2 in comparison with mature human skin mast cells—Drastically reduced levels of tryptase and chymase in mast cell lines. Exp. Dermatol..

[55] Tatemoto K., Nozaki Y., Tsuda R., Konno S., Tomura K., Furuno M., Ogasawara H., Edamura K., Takagi H., Iwamura H. (2006). Immunoglobulin E-independent activation of mast cell is mediated by mrg receptors. Biochem. Biophys. Res. Commun..

[56] Fujisawa D., Kashiwakura J., Kita H., Kikukawa Y., Fujitani Y., Sasaki-Sakamoto T., Kuroda K., Nunomura S., Hayama K., Terui T. (2014). Expression of mas-related gene *X2* on mast cells is upregulated in the skin of patients with severe chronic urticaria. J. Allergy Clin. Immunol..

[57] Subramanian H., Gupta K., Ali H. (2016). Roles of Mas-related G protein-coupled receptor X2 on mast cell-mediated host defense, pseudoallergic drug reactions, and chronic inflammatory diseases. J. Allergy Clin. Immunol..

[58] Gaudenzio N., Sibilano R., Marichal T., Starkl P., Reber L.L., Cenac N., McNeil B.D., Dong X., Hernandez J.D., Sagi-Eisenberg R. (2016). Different activation signals induce distinct mast cell degranulation strategies. J. Clin. Investig..

[59] Kim C.H. (2008). Regulation of FoxP3 regulatory T cells and Th17 cells by retinoids. Clin. Dev. Immunol..

[60] Ertesvåg A., Naderi S., Blomhoff H.K. (2009). Regulation of B cell proliferation and differentiation by retinoic acid. Semin. Immunol..

[61] Artuc M., Steckelings U.M., Grutzkau A., Smorodchenko A., Henz B.M. (2002). A long-term coculture model for the study of mast cell-keratinocyte interactions. J. Investig. Dermatol..

[62] Kim J., Guhl S., Babina M., Zuberbier T., Artuc M. (2014). Integration of the human dermal mast cell into the organotypic co-culture skin model. Methods Mol. Biol..

[63] Babina M., Guhl S., Artuc M., Zuberbier T. (2016). Skin mast cell phenotypes between two highly divergent cohorts—More pronounced variability within than between groups. Exp. Dermatol..

[64] Babina M., Schulke Y., Kirchhof L., Guhl S., Franke R., Bohm S., Zuberbier T., Henz B.M., Gombart A.F. (2005). The transcription factor profile of human mast cells in comparison with monocytes and granulocytes. Cell. Mol. Life Sci..

[65] Harvima I.T., Karkola K., Harvima R.J., Naukkarinen A., Neittaanmaki H., Horsmanheimo M., Fraki J.E. (1989). Biochemical and histochemical evaluation of tryptase in various human tissues. Arch. Dermatol. Res..

